# The Impact of Obesity on *Clostridioides difficile* Infection Outcomes: A Retrospective Cohort Study

**DOI:** 10.3390/jcm14155459

**Published:** 2025-08-03

**Authors:** Alaa Atamna, Manar Khalaila, Tanya Babich, Anan Zriek, Haim Ben Zvi, Gida Ayada, Avishay Elis, Jihad Bishara, Amir Nutman

**Affiliations:** 1 Infectious Diseases Unit, Rabin Medical Center, Beilinson Hospital, 39 Jabotinsky Road, Petah-Tikva 49100, Israel; anan.zr35@gmail.com (A.Z.); jihadb@clalit.org.il (J.B.); 2 Faculty of Medical and Health Sciences, Tel-Aviv University, Tel-Aviv 69978, Israel; manark@mail.tau.ac.il (M.K.); tanyanoa@gmail.com (T.B.); haimbe@clalit.org.il (H.B.Z.); avishayel@clalit.org.il (A.E.); anutman@gmail.com (A.N.); 3 Clinical Research Authority, Rabin Medical Center, Beilinson Hospital, Petah-Tikva 49100, Israel; 4 Clinical Microbiology Laboratory, Rabin Medical Center, Beilinson Hospital, Petah-Tikva 49100, Israel; 5 Internal Medicine C, Rabin Medical Center, Beilinson Hospital, Petah-Tikva 49100, Israel; ghidaa.692@gmail.com; 6 Hospital Management, Wolfson Medical Center, Holon 58100, Israel

**Keywords:** *Clostridioides difficile* infection (CDI), obesity, body mass index (BMI), mortality, outcomes, survival paradox

## Abstract

**Background:** Studies have demonstrated a positive correlation between high body mass index (BMI) and an increased risk of *Clostridioides difficile* infection (CDI), independent of antibiotic usage or healthcare exposures. **Aim:** To compare the outcomes of obese (BMI ≥ 30 kg/m^2^) and non-obese (BMI < 30 kg/m^2^) hospitalized patients with CDI. **Methods:** This retrospective cohort study included patients with CDI hospitalized in Beilinson hospital between January 2013 and January 2020. The primary outcome was 90-day all-cause mortality. Secondary outcomes included 30-day mortality, colectomy, intensive care unit (ICU) admission and length of hospital stay (LOS). Multivariate analysis was performed to identify the risk factors independently associated with 90-day mortality. **Results:** The study included 889 patients: 131 (15%) obese and 758 (85%) non-obese. The obese group was younger (median age 65 years vs. 73 years (*p* < 0.01)) and with a higher rate of diabetes mellitus (57/131 (44%) vs. 180/758 (24%) (*p* < 0.01)). The 90-day mortality was lower in the obese group: 19/131 (15%) vs. 170/752 (23%) (*p* = 0.04). The 30-day mortality was 8/131 (6%) vs. 96/757 (13%) (*p* = 0.03). ICU admission was 9/131 (7%) vs. 23/758 (3%) (*p* = 0.03), and median LOS was 19 vs. 12 days (*p* < 0.01) in obese and non-obese groups, respectively. In the multivariable analysis, after adjustment for age, Charlson’s comorbidity index ≥3, assistance in activities of daily living, treatment with proton pump inhibitors and severity of illness, obesity was not a significant risk factor for 90-day mortality (OR = 0.65, 95% CI: 0.38–1.01; *p* = 0.1). **Conclusions:** In this study, obesity was not significantly associated with 90-day mortality after adjustment for other risk factors; however, ICU admission was higher and LOS longer in this group.

## 1. Introduction

*Clostridioides difficile* is a spore-forming Gram-positive rod that belongs to the Firmicutes phylum [1]. It is considered the principal cause of infectious diarrhea among hospitalized patients, leading to significant morbidity, prolonged hospital stays and increased healthcare costs [2,3,4,5].

Over the last few decades, the incidence of *C. difficile* infection (CDI) has been rising globally, with an increased burden on both healthcare and community settings [6,7]. In Israel, the National Center for Infection Control reported that the incidence of CDI increased from 49.9 cases per 100,000 patient days in 2019 to 56.9 per 100,000 patient days in 2024, reflecting a 14% rise [8].

One of the main risk factors for *Clostridioides difficile* infection (CDI) is exposure to antimicrobial agents, which disrupt the intestinal microbiota, leading to an increased Firmicutes/Bacteroidetes ratio [9,10,11]. This dysbiosis depletes protective species that normally inhibit the growth of *C. difficile*. This disruption creates an environment where *C. difficile* can proliferate, facilitated by its resistance to many antibiotics [12].

The global obesity epidemic has emerged as a significant public health challenge, contributing to the rise of preventable diseases, such as cardiovascular disease, diabetes mellitus and certain cancers [13]. Beyond these well-documented health risks, obesity also influences the gut microbiota, often reducing microbial diversity and altering specific bacterial populations. The Firmicutes/Bacteroidetes ratio has been studied as a key indicator of obesity-related dysbiosis in both adults and children [14]. A systematic review of 32 studies comparing the gut microbiota profiles of obese and lean individuals found that obese patients exhibit a higher Firmicutes/Bacteroidetes ratio [15], similar to the shifts in microbiota composition observed in antimicrobial-associated dysbiosis. This imbalance may predispose them to *C. difficile* colonization and infection, as highlighted in a recent review [16].

Research indicates that host inflammation influences CDI outcomes [17]. Obesity is characterized by elevated levels of pro-inflammatory cytokines, adipokines and other mediators, which alter immune function [18]. Thus, in addition to microbiota changes that predispose individuals to CDI, the inflammatory environment in obesity may further contribute to more severe or prolonged infections.

Several studies have reported a positive correlation between obesity and an increased risk of CDI, independent of antibiotic use or healthcare exposures. Bishara et al. found that high body mass index (BMI) was a significant risk factor for CDI in hospitalized patients (odds ratio (OR) = 1.19, 95% confidence interval (CI): 1.12–1.27) [19]. Similarly, Leung et al. reported a positive association between obesity and CDI (OR = 4.06, 95% CI: 1.15–14.36) [20]. However, other studies have shown contradictory results. A recent meta-analysis found that high BMI was associated with decreased odds of CDI (pooled OR = 0.88, 95% CI: 0.8–0.97) [21]. Additionally, studies examining the relationship between obesity and CDI severity have yielded mixed results. Mulki et al. found that individuals with a BMI > 35 kg/m^2^ were 1.7 times more likely to develop severe CDI compared to those with a BMI between 20 and 35 kg/m^2^ [22]. In contrast, Chatterjee et al. did not find a significant association between obesity and severe CDI (adjusted OR = 1.12, 95% CI: 0.59–2.15) [23].

These conflicting results highlight the need for further investigation into the role of obesity in CDI. Our study aims to address this gap by comparing clinical outcomes in obese and non-obese hospitalized adult patients with CDI to clarify the impact of obesity on CDI outcomes and inform clinical decision making, including risk stratification and preventive measures.

## 2. Methods

### 2.1. Study Setting and Population

The study was conducted at Beilinson hospital, an 830-bed tertiary care center in Petach Tikva, Israel. We included all consecutive adult inpatients with an episode of CDI between January 2013 and January 2020. Both community and hospital onset CDI episodes were included. Patients were included in the study only for their first episode of CDI during the study period, and any subsequent episodes were excluded from the analysis. The study protocol was approved by the Rabin Medical Center Ethics Committee (RMC-0987-20) (date of approval: 9 February 2022). Informed consent was waived due to the retrospective nature of the study.

### 2.2. Definitions

Obesity was defined as BMI ≥ 30 kg/m^2^. 

A CDI episode was defined as at least 3 unformed stools within 24 h along with a positive microbiological assay, as described below [24,25]. 

Severe CDI was defined according to the Infectious Diseases Society of America (IDSA) guidelines [24] as white blood cell (WBC) count ≥ 15,000 cells/mL or creatinine level higher than 1.5 mg/dL.

### 2.3. Outcomes

The primary outcome was 90-day all-cause mortality. Secondary outcomes included 30-day all-cause mortality, ICU admission, the need for colectomy and length of hospital stay (LOS) following CDI diagnosis.

### 2.4. Microbiological Methods

Laboratory diagnosis was made according to the Israeli Ministry of Health guidelines using a modified two-step algorithm [25]. First, a rapid membrane enzyme immunoassay (EIA) was used for the simultaneous detection of *C. difficile* glutamate dehydrogenase (GDH) antigen and toxins A and B in fecal specimens (C. DIFF QUICK CHEK COMPLETE^®^, TECHLAB^®^, Blacksburg, VA, USA). The assay was considered positive when both GDH and toxin A/B antigens were positive. In cases where there were discrepant results in the initial assay, a confirmatory PCR test was performed to detect the presence of C. difficile toxin genes (Xpert^®^ C. difficile, Cepheid, Sunnyvale, CA, USA).

### 2.5. Data Collection

Data were collected from the electronic medical records and the microbiological laboratory computerized system. The data included patient demographics (age, sex, assistance in activity of daily living (ADL) and nursing home residency), BMI, chronic comorbidities (hypertension, diabetes mellitus, chronic kidney disease, malignancies—solid and hematologic, inflammatory bowel disease, chronic liver disease, solid organ transplantation and hematopoietic stem cell transplantation), treatment with a proton pump inhibitor (PPI), the positive microbiological assay (EIA or PCR), vital signs at infection presentation (temperature and systolic blood pressure), laboratory results at infection presentation (WBC count, creatinine, albumin and C-reactive protein (CRP) levels) and CDI antibiotic treatment. Charlson comorbidity index (CCI) was calculated to assess the overall burden of comorbidities [26].

### 2.6. Statistical Analyses

We compared the characteristics and outcomes between obese and non-obese patients with CDI. Data are presented as the mean ± standard deviation or median and interquartile range (IQR) for continuous variables and the number of subjects and percentage for categorical variables. All variables were tested for normality using the non-parametric Kolmogorov–Smirnov test. Continuous variables were compared using Student’s *t*-test for normally distributed variables and Wilcoxon rank sum for skewed variables. Categorical variables were compared using the χ^2^ test or Fisher’s exact test. Multiple imputation was used to handle missing data.

Multivariable logistic regression analysis was performed to identify factors independently associated with 90-day mortality. Variables with *p* ≤ 0.1 in univariate analyses were considered for inclusion in the multivariable model. After model fitting and assessment using the Akaike information criterion (AIC) [27], only those variables that contributed to the best-fitting model were retained for the final analysis. We reported ORs with 95% CIs to quantify the strength of associations. Statistical analysis was performed using IBM SPSS software v.28.0.

## 3. Results

A total of 889 patients were included in the study. Of them, 131 (15%) were obese and 758 (85%) non-obese. Table 1 presents the baseline characteristics of the study cohort. The obese group was younger (median age 65 years vs. 73 years, *p* < 0.01) and had fewer comorbidities (CCI ≥ 3 in 82/131 (63%) vs. 547/758 (72%), *p* = 0.03); however, this group had a higher rate of diabetes mellitus (57/131 (44%) vs. 180/758 (24%), *p* < 0.01).

Table 2 presents the clinical characteristics of the CDI episode. At the time of CDI onset, systolic blood pressure was higher in the obese group (127 mmHg vs. 118 mmHg, *p* < 0.01), while the WBC count was lower (9390 cells/µL vs. 11,600 cells/µL, *p* = 0.01) compared to the non-obese group. The rate of severe CDI was similar (80/119 (67%) vs. 510/695 (73%), *p* = 0.2) between groups. Vancomycin was the most frequently prescribed antibiotic in approximately two-thirds of patients in both groups (Table 2).

CDI outcomes are presented in Table 3. The primary outcome, 90-day mortality, was lower in the obese group (19/131 (15%) vs. 170/752 (23%), *p* = 0.04). Regarding secondary outcomes, ICU admission rate was more than double in the obese group (9/131 (7%) vs. 23/758 (3%), *p* = 0.03), and the LOS was significantly longer (median 19 days vs. 12 days, *p* < 0.01).

The risk factors for 90-day mortality are presented in Table 4. In the univariate analysis, patients who died within 90 days were less likely to be obese (19/189 (10%) vs. 112/694 (16%), *p* = 0.04), older (median age 77 vs. 70 years, *p* < 0.01), needed assistance in ADL (105/189 (56%) vs. 288/694 (42%), *p* < 0.01), had more comorbidities (CCI ≥ 3, 165/189 (87%) vs. 461/694 (66%), *p* < 0.01), severe CDI (145/182 (80%) vs. 444/631 (70%), *p* = 0.01), hypoalbuminemia (86/156 (55%) vs. 230/556 (41%), *p* < 0.01) and were treated with PPIs (93/189 (49%) vs. 274/694 (40%), *p* = 0.02).

For the multivariable analysis, five models with different subsets of covariates were tested using variables with *p* ≤ 0.1 from the univariate analysis. The model with the best fit, selected based on the lowest AIC, was chosen for the final analysis and included the following variables (Table 5): obesity, severe CDI, CCI ≥ 3, PPI treatment and age. Among these, only CCI ≥ 3 was a significant predictor of 90-day mortality (OR 2.42, 95% CI: 1.40–4.18). Obesity, however, was not a significant risk factor for mortality after adjustment for other variables in the model (OR = 0.65, 95% CI: 0.38–1.01; *p* = 0.1).

## 4. Discussion

Our study found that obese patients had a higher rate of ICU admission and longer LOS but a lower rate of 90-day mortality. However, after adjusting for other risk factors, the association between obesity and 90-day mortality was not statistically significant.

There are conflicting results in the medical literature regarding the relationship between obesity and mortality following CDI. A large retrospective study of 3851 CDI hospitalized patients from three medical centers in the USA between 2010 and 2018 did not find a significant association between obesity and 30-day mortality (OR = 0.96, 95% CI: 0.69–1.34 for BMI ≥ 30 kg/m^2^ vs. 20–30 kg/m^2^) [28]. In a retrospective study that analyzed 22,937 emergency department visits across the USA, only extremely high BMI was associated with mortality: the adjusted ORs for in-hospital mortality were 2.71 (95% CI: 1.51–4.84) and 3.07 (95% CI 1.61–5.84) for BMI 40–44.9 kg/m^2^ and BMI 45–49.9 kg/m^2^, respectively, as compared with normal BMI (19–24.9 kg/m^2^) [29]. In our cohort, only 18 (2%) patients had a BMI ≥ 40 kg/m^2^; therefore, we could not analyze this group separately.

Other studies found lower mortality in obese patients with CDI. Tariq et al. conducted a nationwide retrospective study of approximately 920,000 inpatients with CDI in the USA, which found that obesity was associated with a decreased risk of mortality (OR = 0.76, 95% CI 0.70–0.81). The protective effect remained significant in patients who were morbidly obese [30]. Another retrospective study from the USA by Jaikumar et al. that included 89,649 patients with CDI found that the unadjusted mortality was lower in obese patients compared to non-obese patients; however, this association was not statistically significant (OR 0.71, 95% CI: 0.38–1.33) [31].

The lower mortality, often referred to as the “obesity survival paradox” in CDI, may be explained by the greater metabolic reserve in obese patients compared to those with normal weight, potentially conferring increased resilience to the heightened catabolism during infection. In addition, obese patients may present more “care-seeking” behavior because they have more comorbidities, increasing the chance of being diagnosed and hospitalized with CDI.

The gut microbiota can play a protective role in certain infections. For example, an experimental study in mice identified the intestinal microbiota as a protective mediator in pneumococcal pneumonia by enhancing primary alveolar macrophage function [32]. Similarly, the gut microbiota has been shown to modulate the severity of CDI. Specifically, the relative abundance of genera such as *Akkermansia*, *Anaerotignum*, *Blautia*, *Enterocloster*, *Lactonifactor* and *Monoglobus* has been linked to decreased toxin production, lower histopathological scores of cecal tissue and reduced mortality in a mouse model [33]. In contrast, obesity-related dysbiosis, characterized by an increased Firmicutes/Bacteroidetes ratio, may predispose individuals to CDI. Bacteria from the Firmicutes phylum metabolize primary bile acids into secondary bile acids, and a reduction in these bacteria leads to lower secondary bile acid levels. This decrease diminishes the inhibition of *C. difficile* germination, potentially facilitating CDI development [16]. Further research is needed to clarify how obesity-related dysbiosis influences CDI outcomes, particularly in light of the negative findings from clinical studies. Investigating microbial imbalances beyond the Firmicutes/Bacteroidetes ratio, including the role of specific bacterial taxa or gut-derived metabolites, could provide deeper insights into the mechanisms linking obesity and CDI.

A small proportion of our study cohort consisted of immunocompromised oncologic and transplant recipients, whose gut microbiota may have been altered by chemotherapy and immunosuppressive drugs. Chemotherapy in particular impacts microbial diversity by decreasing Bacteroidetes and increasing taxa such as Clostridiaceae and Streptococcaceae, which can further influence infection risks [34,35].

Obese patients had a higher rate of ICU admission and longer length of stay (LOS). However, confounding factors cannot be ruled out. Since these were secondary outcomes, we did not perform a multivariable analysis to control for potential confounders.

The strengths of our study include the large cohort and consecutive enrollment of all hospitalized CDI cases during the study period, as well as detailed information from electronic medical records, which allowed adjustment for confounders. Our study had several limitations. First, it is a single-center study, limiting the applicability of our findings to other centers. Second, the retrospective nature of the study makes it vulnerable to information bias. Third, because of the small number of patients with extremely high BMI (≥40 kg/m^2^), we were unable to analyze its impact on mortality. Future research should evaluate the association between obesity and CDI recurrence to further clarify its potential impact on long-term outcomes. Finally, understanding the potential protective or exacerbating role of the immune response in obese individuals with CDI warrants further exploration.

In conclusion, while obesity was not independently associated with 90-day mortality after adjusting for other risk factors, it was associated with worse clinical outcomes, including higher ICU admission rates and longer LOS. These findings suggest that obesity may complicate the clinical management of CDI. Given the growing global burden of both obesity and CDI, more research is needed to better understand how obesity influences CDI outcomes, which could lead to improved patient management and care strategies in this population.

## Figures and Tables

**Table 1:** Baseline characteristics of obese (BMI ≥ 30 kg/m^2^) and non-obese (BMI < 30 kg/m^2^) hospitalized patients with CDI.

Variable	Entire Cohort (*n* = 889)	BMI ≥ 30 kg/m^2^ (*n* = 131)	BMI < 30 kg/m^2^ (*n* = 758)	*p*-Value
Age, years, median (IQR)	71 (59–83)	65 (56–79)	73 (60–84)	<0.01
BMI, kg/m^2^, median (IQR)	24.4 (21.3–27.3)	33.3 (31.2–37.1)	23.6 (20.7–25.5)	<0.01
Female gender, n/total n. (%)	460/888 (52%)	69/131 (53%)	391/757 (52%)	0.8
CCI, median (IQR)	5 (3–7)	5 (3–7)	5 (3–7)	0.4
CCI ≥ 3 points, n (%)	629 (71%)	82 (63%)	547 (72%)	0.03
Assistance in ADL, n (%)	395 (44%)	51 (39%)	344 (45%)	0.2
Nursing home residency, n (%)	138/887 (16%)	18 (14%)	120/756 (16%)	0.5
Comorbidities:				
Hypertension, n (%)	400 (45%)	62 (47%)	338 (45%)	0.6
Diabetes mellitus, n (%)	237 (27%)	57 (44%)	180 (24%)	<0.01
Chronic kidney disease, n (%)	115 (13%)	17 (13%)	98 (13%)	0.9
Liver disease, n (%)	21 (2%)	6 (5%)	15 (2%)	0.07
Inflammatory bowel disease, n (%)	31 (4%)	1 (0.8%)	30 (4%)	0.07
Solid tumor, n (%)	131 (15%)	24 (18%)	107 (14%)	0.2
Hematologic malignancy, n (%)	84 (9%)	9 (7%)	75 (10%)	0.3
Solid organ transplant, n (%)	62 (7%)	8 (6%)	54 (7%)	0.7
HSC transplant, n (%)	29 (3%)	4 (3%)	25 (3%)	1
PPI treatment, n (%)	369 (42%)	59 (45%)	310 (41%)	0.4

IQR—interquartile range; BMI—body mass index; CCI—Charlson comorbidity index; ADL—activity of daily living; HSC—hematopoietic stem cells; PPI—proton pump inhibitor.

**Table 2:** Clinical characteristics of obese (BMI > 30 kg/m^2^) and non-obese (BMI < 30 kg/m^2^) patients at onset of CDI episode.

Variable	Entire Cohort (*n* = 889)	BMI ≥ 30 kg/m^2^ (*n* = 131)	BMI < 30 kg/m^2^ (*n* = 758)	*p*-Value
Vital signs				
Temperature, C°, median (IQR)	36.9 (36.6–37.4)	36.8 (36.6–37.3)	36.9 (36.6–37.5)	0.08
Systolic blood pressure, mmHg, median (IQR)	119 (105–138)	127 (112–141)	118 (103–138)	<0.01
Laboratory results				
WBC count, 10^9^ cells/L, median (IQR)	11.2 (6.72–17.3)	9.39 (6.55–14.1)	11.6 (6.8–17.4)	0.01
Creatinine level, mg/dL, median (IQR)	1.05 (0.73–1.65)	1.04 (0.76–1.73)	1.05 (0.72–1.64)	0.5
Creatinine level > 1.5 mg/dL, n (%)	248/864 (29%)	39/129 (30%)	209/735 (28%)	0.7
Albumin, g/dL, mean ± SD	3.18 ± 0.67	3.28 ± 0.71	3.16 ± 0.67	0.1
Albumin ≤ 3 g/dL, n/total n (%)	316/718 (44%)	41/105 (39%)	275/613 (45%)	0.3
CRP, mg/dL, median (IQR)	6.9 (2.67–15.14)	6.6 (2.86–13.7)	7.2 (2.62–15.3)	0.8
CRP ≥ 10 mg/dL, n/total n (%)	245/639 (38%)	34/96 (35%)	211/543 (39%)	0.5
Microbiological diagnosis by PCR, n (%)	485 (55%)	73 (56%)	412 (54%)	0.8
Severe CDI *	590/814 (72%)	80/119 (67%)	510/695 (73%)	0.2
CDI treatment				
Vancomycin, n (%)	554 (62%)	87 (66%)	467 (62%)	0.3
Metronidazole, n (%)	258 (29%)	30 (23%)	228 (30%)	0.09
Fidaxomicin, n (%)	80 (9%)	14 (11%)	66 (9%)	0.5
Tigecyclin, n (%)	27 (3%)	3 (2%)	24 (3%)	0.8

CDI—*Clostridioides difficile* infection; BMI—body mass index; IQR—interquartile range; SD—standard deviation; WBC—white blood cells; CRP—C-reactive protein. * Severe CDI diagnosis was made if white blood cell count was ≥15,000 cells/mL or creatinine level was higher than 1.5 mg/dL.

**Table 3:** Outcomes of obese (BMI > 30 kg/m^2^) and non-obese (BMI < 30 kg/m^2^) patients with CDI.

Variable	BMI ≥ 30 kg/m^2^ (*n* = 131)	BMI < 30 kg/m^2^ (*n* = 758)	*p*-Value
90-day mortality, n/total n (%)	19/131 (15%)	170/752 (23%)	0.04
30-day mortality, n/total n (%)	8/131 (6%)	96/757 (13%)	0.03
ICU admission, n (%)	9 (7%)	23 (3%)	0.03
Colectomy, n (%)	2 (1.5%)	6 (0.8%)	0.3
Length of hospital stay, days, median (IQR)	19 (7–33)	12 (6–25)	<0.01

CDI—*Clostridioides difficile* infection; BMI—body mass index; ICU—intensive care unit; IQR—interquartile range.

**Table 4:** Univariate analysis of risk factors for 90-mortality in hospitalized patients with CDI.

Variable	90-Day Mortality		*p*-Value
	Yes (*n* = 189)	No (*n* = 694)	
Age, years, median (IQR)	77 (65–86)	70 (57–82)	<0.01
Female gender, n (%)	95 (50%)	362 (52%)	0.6
BMI, kg/m^2^, median (IQR)	23.4 (20.6–26)	24.9 (21.6–28)	0.008
Obesity (BMI ≥ 30 kg/m^2^)	19 (10%)	112 (16%)	0.04
CCI, median (IQR)	6 (4–8)	5 (3–7)	<0.01
CCI ≥ 3 points, n (%)	165 (87%)	461 (66%)	<0.01
Assistance in ADL, n (%)	105 (56%)	288 (42%)	<0.01
Nursing home residency, n (%)	31 (16%)	106 (15%)	0.7
Hypertension, n (%)	94 (50%)	305 (44%)	0.2
Diabetes mellitus, n (%)	54 (29%)	182 (26%)	0.5
Liver disease, n (%)	5 (3%)	16 (2%)	0.8
Inflammatory bowel disease, n (%)	0 (0%)	29 (4%)	0.002
Chronic kidney disease, n (%)	28 (15%)	86 (12%)	0.4
Solid tumors, n (%)	46 (24%)	84 (12%)	<0.01
Hematologic malignancy, n (%)	16 (9%)	67 (10%)	0.6
Solid organ transplant, n (%)	6 (3%)	56 (8%)	0.02
HSCT, n (%)	7 (4%)	21 (3%)	0.6
PPI treatment, n (%)	93 (49%)	274 (40%)	0.02
WBC count, 10^9^ cells/L, median (IQR)	12.1 (6.8–19.6)	10.9 (6.7–16.7)	0.06
Creatinine level, mg/dL, median (IQR)	1.11 (0.74–1.77)	1.02 (0.73–1.6)	0.3
Creatinine level > 1.5 mg/dL, n (%)	60/187 (32%)	188/677 (28%)	0.2
Albumin, g/dL, mean ± SD	2.98 ± 0.62	3.23 ± 0.68	<0.01
Albumin ≤ 3 g/dL, n/total n (%)	86/156 (55%)	230/556 (41%)	<0.01
CRP, mg/dL, median (IQR)	7.6 (3.54–0.85)	6.6 (2.53–15.09)	0.3
CRP > 10 mg/dL, n/total n (%)	55 (40%)	188 (38%)	0.6
Microbiological diagnosis by PCR, n (%)	93 (49%)	387 (56%)	0.1
Severe CDI *, n/total n (%)	145/182 (80%)	444/631 (70%)	0.01
Colectomy, n (%)	4 (2.1%)	4 (0.6%)	0.05
ICU admission, n (%)	12 (6%)	20 (3%)	0.02
CDI treatment			
Metronidazole, n (%)	59 (31%)	199 (29%)	0.5
Vancomycin, n (%)	109 (58%)	439 (63%)	0.2
Fidaxomicin, n (%)	19 (10%)	60 (9%)	0.5
Tigecyclin, n (%)	7 (4%)	20 (3%)	0.6

CDI—*Clostridioides difficile* infection; IQR—interquartile range; BMI—body mass index; CCI—Charlson comorbidity index; ADL—activity of daily living; ICU—intensive care unit; WBC—white blood cells; CRP—C-reactive protein; HSCT—hematopoietic stem cell transplant; PPI—proton pump inhibitor. * Severe CDI diagnosis was made if white blood cell count was ≥15,000 cells/mL or creatinine level was higher than 1.5 mg/dL.

**Table 5:** Multivariate model for risk factors of 90-day mortality in hospitalized patients with CDI.

Risk Factor	Adjusted OR (95% CI)	*p*-Value
Obesity (BMI ≥ 30 kg/m^2^)	0.65 (0.38–1.01)	0.1
Severe CDI * vs. non-severe	1.47 (0.98–2.19)	0.06
CCI ≥ 3 vs. <3	2.42 (1.40–4.18)	<0.01
PPI treatment	1.35 (0.96–1.88)	0.08
Age (years)	1.01 (0.99–1.02)	0.2
Assistance in ADL	1.19 (0.83–1.72)	0.3

CDI—*Clostridioides difficile* infection; BMI—body mass index; PPI—proton pump inhibitor; ADL—activity of daily living; OR—odds ratio; CI—confidence interval. * Severe CDI diagnosis was made if white blood cell count was ≥15,000 cells/mL or creatinine level was higher than 1.5 mg/dL.

## Data Availability

The datasets used and/or analyzed in the current study are available from the corresponding author upon reasonable request.
